# White Shark Optimization for Solving Workshop Layout Optimization Problem

**DOI:** 10.3390/biomimetics10050268

**Published:** 2025-04-27

**Authors:** Bin Guo, Yuanfei Wei, Qifang Luo, Yongquan Zhou

**Affiliations:** 1 College of Artificial Intelligence, Guangxi Minzu University, Nanning 530006, China; 202220812002004@stu.gxmzu.edu.cn; 2 Faculty of Information Science and Technology, Universiti Kebangsaan Malaysia—UKM, Bangi 43600, Selangor, Malaysia; weiyuanfei@gxxshxy.edu.cn; 3 Guangxi Key Laboratories of Hybrid Computation and IC Design Analysis, Nanning 530006, China

**Keywords:** White Shark Optimizer, workshop layout, intelligent optimization

## Abstract

The workshop is a crucial site for ensuring the smooth operation of production activities within an enterprise, playing a significant role in its long–term development. A well–designed workshop layout can reduce material–handling costs during production and enhance the overall efficiency of the enterprise. This paper establishes a mathematical model for the workshop layout problem, aiming to minimize logistics transportation costs and maximize non–logistics relationships. Using a real–world case study, the White Shark Optimizer (WSO) algorithm is applied to solve the model. The results show that the transportation distance of the layout scheme obtained by the WSO algorithm is reduced by 381 m, 82 m, and 56 m, respectively, compared with the original layout, the Genetic Algorithm (GA), and the Sparrow Search Algorithm (SSA), and the non–logical relationship is increased by 24.84% and 1.6%, respectively. The layout scheme obtained by using the WSO algorithm is more excellent and can effectively improve the production efficiency of enterprises.

## 1. Introduction

With the gradual expansion of personalized market demands, competition within the manufacturing sector has become increasingly fierce. The manufacturing industry serves as a reflection of a nation’s comprehensive strength and holds critical significance for its long–term development [1,2,3]. To meet diverse customer demands, enterprises have invested heavily in improving product and service quality, resulting in a significant increase in production costs. As a critical hub for ensuring smooth production operations, workshops hold substantial importance in achieving efficient and precise manufacturing processes, both in terms of technological applications and engineering implementation. Data from manufacturing enterprises reveal that a considerable portion of costs is attributed to material handling, where prolonged waiting times during transportation reduce employee efficiency, leading to substantial operational waste. A well–designed and scientific workshop layout can effectively reduce production costs, enhance profitability, and support long–term enterprise development. As a pivotal challenge for manufacturing enterprises, an optimized workshop layout not only minimizes material–handling costs but also improves production efficiency [4,5,6].

Company D is an enterprise engaged in the manufacturing of solid waste treatment equipment. In recent years, this enterprise has adjusted its production content in response to changes in market demand and product structure. However, its workshop layout has always been based on the original layout and has not been optimized according to production demands, resulting in a disconnection between the workshop distribution and logistics transportation routes and the existing production processes. The original layout of the enterprise led to a low efficiency in material handling, resulting in a decline in the overall production efficiency of the enterprise. In order to improve the production efficiency of the enterprise, the overall layout of the workshop needs to be optimized. By adjusting the workshop locations and optimizing the logistics routes, the production smoothness can be improved and the market competitiveness of the enterprise can be enhanced.

Metaheuristic algorithms have been widely utilized to solve various types of complex optimization problems in both academia and industry, and numerous experiments have demonstrated their significant practical value in addressing such challenges. These algorithms operate by following specified search mechanisms and strategies to identify optimal or near–optimal solutions within the search space. Exploration and exploitation are two fundamental search strategies in metaheuristic algorithms. Exploration employs a macro–level search to locate new regions of potential solutions, while exploitation utilizes a micro–level search to gather critical information from identified search areas. Due to their simple principles, ease of understanding, and straightforward implementation, these algorithms have garnered increasing attention, such as the Genetic Algorithm (GA) [7], Differential Evolution (DE) [8], Particle Swarm Optimization (PSO) [9], Simulated Annealing (SA) [10], the Whale Optimization Algorithm (WOA) [11], and Ant Colony Optimization (ACO) [12], among others. In this paper, the White Shark Optimization (WSO) algorithm is employed to solve the workshop layout problem.

The rest of this paper is as follows: Section 2 is the related work introduction. Section 3 presents the mathematical model of the workshop layout problem. Section 4 is the experimental results and discussion. Section 5 is the conclusion.

## 2. Related Work

### 2.1. White Shark Optimizer

The White Shark Optimizer (WSO) algorithm is a new swarm intelligence optimization algorithm proposed by Malik Braik et al. in 2022 [13]. Its inspiration stems from the process by which great white sharks search for food and track prey in the deep sea. Great white sharks are highly adaptable predators and remarkable hunters, possessing powerful muscles, keen eyesight, and a sharp sense of smell. Firstly, white sharks can detect the waves generated by the movement of prey and use their unique auditory and olfactory senses to search for and move toward the prey. Secondly, they randomly search for prey in the ocean and continuously approach the best target. Finally, they move based on the behavior of the fish swarm. The White Shark Optimizer (WSO) is mathematically modeled based on the hunting strategies of great white sharks, featuring strong optimization capabilities and fast convergence speed.

In the White Shark Optimizer (WSO), the position of an individual shark can be represented as $w_{i}=(w_{1},w_{2}\cdots ,w_{n})$, where n denotes the dimensionality of the problem to be solved. Great white sharks spend most of their time hunting and tracking prey. They can sense the location of prey by detecting the waves generated by the prey’s movement. At this point, the shark moves toward the prey in an undulating motion, and the equations are as follows:

(1) $$v_{k+1}^{i}=\mu [v_{k}^{i}+p_{1}(w_{gbest_{k}}-w_{k}^{i})\times c_{1}+p_{2}(w_{best}^{v_{k}^{i}}-w_{k}^{i})\times c_{2}]$$

where i represents the index of the individual white shark, k denotes the iteration count, $w_{gbest_{k}}$ indicates the best position found by the white shark population in the k–th iteration, and $w_{best}^{v_{k}^{i}}$ represents the optimal position found by the i–th individual in the k–th iteration. μ is the contraction factor for the movement speed of the white shark, and both $p_{1}$ and $p_{2}$ are dynamically adjusted based on the iteration count, changing as the iterations progress. $c_{1}$ and $c_{2}$ are random numbers between 0 and 1.

When a white shark detects the scent of prey or the waves generated by the movement of prey, it will move closer to the location of the prey. By the time the white shark reaches the location, the prey may have already moved away from its original position. However, the prey leaves behind a scent at the original location, which the white shark can still detect. Therefore, with regard to the position update of the white shark, the equations are as follows:

(2) $$w_{k+1}^{i}=\left\{\begin{array}{l} w_{k}^{i}\cdot \neg ⊕w_{o}+u\cdot a+l\cdot b;\;\;\;\;rand<mv\\ w_{k}^{i}+v_{k}^{i}/f;\;\;\;\;\;\;\;\;\;\;\;\;\;\;\;\;\;\;\;\;\;\;\;\;\;\;\;\;\;\;\;rand\geq mv \end{array}\right.$$

(3) $$a=\mathrm{sgn}(w_{k}^{i}-u)>0$$

(4) $$b=\mathrm{sgn}(w_{k}^{i}-l)<0$$

(5) $$w_{o}=⊕(a,b)$$

where $w_{k}^{i}$ represents the i–th individual in the k–th iteration, u and l denote the upper and lower bounds of the search range, $w_{o}$ indicates the positions where the individual $w_{k}^{i}$ exceeds the bounds, the symbol ¬ represents the inversion of this vector, and f denotes the wave frequency. mv represents the strength of the white shark’s sense of smell and hearing, which changes with the number of iterations.

In addition to the undulating motion, white sharks also move toward the best–known position of the prey with a certain probability, and the equations are as follows:

(6) $$w_{k+1}^{`i}=w_{gbest_{k}}+r_{1}{\vec{D}}_{w}\mathrm{sgn}(r_{2}-0.5)\;\;\;\;r_{3}<s_{s}$$

(7) $${\vec{D}}_{w}=\left|rand\times (w_{gbest_{k}}-w_{k}^{i})\right|$$

(8) $$s_{s}=\left|1-e^{\left(-a_{2}\times k/K\right)}\right|$$

(9) $$w_{k+1}^{i}=\frac{w_{k}^{i}+w_{k+1}^{`i}}{2\times rand}$$

where $w_{k+1}^{`i}$ represents the updated position of the white shark relative to the current best position, $\mathrm{sgn}(r_{2}-0.5)$ provides a value of 1 or −1 to change the search direction of the white shark, and $r_{1}$, $r_{2}$, and $r_{3}$ are random numbers between 0 and 1. ${\vec{D}}_{w}$ denotes the distance between the best position and the white shark, and $s_{s}$ represents the sensory intensity of the white shark as it follows the population to approach the prey.

The WSO algorithm balances the exploration and development stages by adjusting the velocity vector and dynamically adjusts the search direction through the olfactory perception mechanism to avoid falling into local optima. Therefore, it has efficient global search and local development capabilities. The adaptive weight factor is introduced, which attenuates dynamically with the number of iterations. There is no need to manually set complex parameters, reducing the difficulty of parameter adjustment. Individual white sharks improve search efficiency by sharing location information, introduce a random disturbance mechanism, increase population diversity, and reduce the risk of premature convergence. The convergence analysis of WSO lacks theoretical support and mainly relies on experimental verification. Parameter design is mostly based on empirical observation and lacks systematic theoretical guidance. Each iteration requires the calculation of the distances and speed updates among multiple individuals. When dealing with high–dimensional problems, the computational burden increases significantly.

### 2.2. Workshop Layout

The conceptual theory of the workshop layout began to emerge in the early 20th century. With the advent of machinery, manual labor was gradually replaced, leading to a significant increase in production efficiency. Manufacturing enterprises also started to pay attention to the design of workshop layouts, basing their layouts on accumulated work experience [14]. In 1961, Muther introduced the Systematic Layout Planning (SLP) method, which provided a standardized procedure for workshop layout planning [15]. This method made the study of workshop layout problems more scientific and rational. Subsequently, various scholars have continued to refine and optimize the SLP method [16,17,18]. Fahad et al. combined the application of lean tools and SLP methods through a case of a wastewater discharge plant, designed a layout plan, and reduced energy consumption and minimized waste management [19]. Bhuvanesh et al. took the automotive parts assembly workshop as the research object, formulated the workshop optimization scheme based on the SLP method, and then evaluated the designed layout optimization scheme through TOPSIS and COPRAS [20]. In analyzing the facility layout problem of the manufacturing system, El–Baz optimized the design based on the Genetic Algorithm and verified the rationality of the scheme with examples [21]. Su Sd et al. designed the layout of the cabin by combining the SLP method with the improved Genetic Algorithm, and the material transportation time was significantly shortened [22]. Nguyen et al. obtained feasible solutions based on the FBCO algorithm by adjusting the parameters. For different types of facilities, the design of the optimal layout scheme is found for facilities with the lowest cost, noise pollution, and safety risk [23]. Gonzalez–Cruz et al. solved the mixed workshop layout problem based on the entropy optimization algorithm to improve the material–handling efficiency [24]. Garcia–Hernandez et al. even proposed an evolutionary algorithm for Coral Reef Optimization, which demonstrated outstanding performance in solving the problem of the facility layout of unequal areas [25]. Korde et al. conducted a simulation design for the manufacturing workshop layout of the wiper pivot plate using Flexsim software, reducing the material–handling time and lowering the material–handling cost [26]. The research contents of these works of literature are shown in Table 1.

In conclusion, through the continuous exploration and improvement of scholars, the solution to layout problems has evolved from simple planning and arrangement to the application of SLP, and then developed to the use of meta–heuristic algorithms for layout analysis based on factors such as human arrangement existing in SLP. With the mature application of computers, various pieces of computer simulation software have also been widely used in the design of layout problems and the verification of scheme effects. Although the research of scholars at home and abroad is increasingly abundant, in real life, many enterprises still make layouts based on the experience of designers. In this regard, WSO is proposed to optimize the actual layout plan of the company, making the layout plan more feasible and applicable. The improvement before and after the optimization of the workshop layout is verified through the total material–handling cost and non–logistics relationship.

Workshop layout optimization involves the combinatorial arrangement of work units within a workshop to derive a suitable layout plan, which is a classic quadratic assignment problem [27,28,29]. To solve the workshop layout problem using intelligent algorithms, it is first necessary to construct an appropriate mathematical model and then utilize intelligent algorithms to solve the model, thereby obtaining an optimized workshop layout. When addressing this problem, practical application cannot be overlooked; the layout must comply with regulations. Factors such as the varying areas of work units, the appropriate distances between them, and the selection of the objective function can directly impact the overall effectiveness of the workshop layout [30]. Therefore, when constructing the model, special attention should be paid to whether the modeling conditions are sufficient and whether the modeling requirements are met. It is crucial that we clearly identify the constraints and understand the relationships between independent and dependent variables. The ultimate goal of workshop layout optimization is to facilitate both material and non–material relationships between work units. When building the mathematical model, it is essential that we ensure that the model is solvable and can yield effective solutions.

## 3. Methodology

### 3.1. Building Monomer Combination Model

In the workshop layout, all work units need to be distributed throughout the workshop. The layout should be realistic and the model should meet the following assumptions:

(1) Coordinate system setting assumption. Take the point at the lower left corner of the workshop as the origin of the rectangular coordinate system. The positive half of the X–axis represents the long side direction of the entire workshop area, and the positive half of the Y–axis serves as the wide side direction of the workshop. This assumption, through the standard Cartesian coordinate system, avoids complex coordinate transformations and is applicable to rectangular or nearly rectangular workshops.
(2) Floor plan assumption. All work units are on the same plane with no height differences, which conforms to the working scenarios of most enterprises. This assumption cannot optimize the logistics transportation in the three–dimensional direction.
(3) Hypothesis of the shape of the working unit. Simplify the shape of each working unit to a rectangle, and stipulate that each rectangular edge is parallel to the *X*–axis and *Y*–axis. This assumption does not take into account the shapes of working units such as circles, which may lead to the waste of space.
(4) Hypothesis of the entrance and exit of the work unit. The entry and exit points of each working unit are the center points of the rectangle. ($x_{i}$,$y_{i}$) represents the coordinates of the center point of working unit *i*, and ($x_{j}$,$y_{j}$) represents the coordinates of the center point of working unit *j*. This assumption takes into account the transportation distances between each workshop and may deviate from the actual transportation distances.

**Figure 1:**
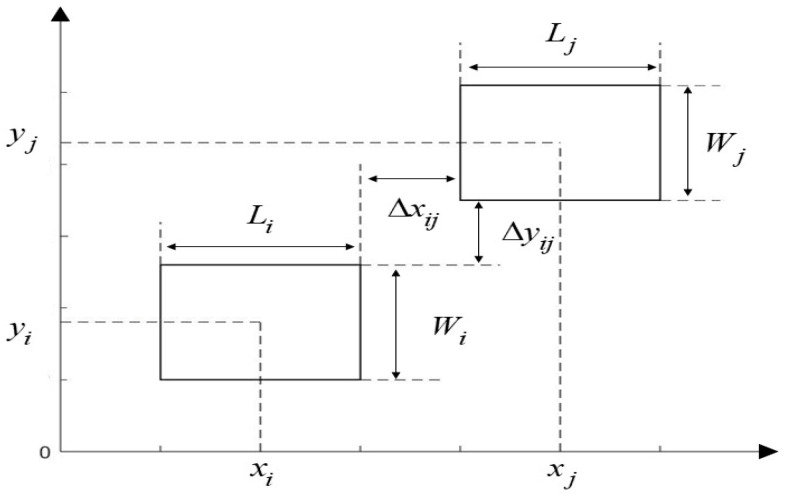
Planar diagram of work units.

Based on the above assumptions, the unit of work relationship diagram is shown in Figure 1. Where i and j represent different units of work. $x_{i}$ and $y_{i}$ represent the center points of the unit of work i. $x_{j}$ and $y_{j}$ represent the center points of the unit of work j. L and W represent the length and width of the work unit. $\Delta x_{ij}$ represents the horizontal distance between the work units. $\Delta y_{ij}$ represents the vertical distance between the work units.

### 3.2. Objective Function

The goal of workshop layout optimization is to reduce the cost of material handling and enhance the degree of connection between work units. Assume that, in a workshop, i and j are the work units of the scheme, respectively. Then, $f_{i,j}$ is used to represent the flow rate between i and j. $c_{i,j}$ represents the moving cost between i and j. $d_{i,j}$ represents the distance between i and j, which adopts the Manhattan distance, and can be calculated as follows:

(10) $$d_{i,j}=\left|x_{i}-x_{j}\right|+\left|y_{i}-y_{j}\right|$$

(11) $$f_{i,j}=\left[\begin{array}{l} f_{11}\;\;\;\;f_{12}\;\;\;\;\cdots \;\;\;\;f_{1m}\\ f_{21}\;\;\;\;f_{22}\;\;\;\;\cdots \;\;\;\;f_{2m}\\ \;\;\vdots \;\;\;\;\;\;\;\vdots \;\;\;\;\ddots \;\;\;\;\;\;\;\;\vdots \\ f_{m1}\;\;\;\;f_{m2}\;\;\cdots \;\;f_{mm} \end{array}\right]$$

(12) $$d_{i,j}=\left[\begin{array}{l} d_{11}\;\;\;\;d_{12}\;\;\;\;\cdots \;\;\;\;d_{1m}\\ d_{21}\;\;\;\;d_{22}\;\;\;\;\cdots \;\;\;\;d_{2m}\\ \;\;\vdots \;\;\;\;\;\;\;\vdots \;\;\;\;\ddots \;\;\;\;\;\;\;\;\vdots \\ d_{m1}\;\;\;\;d_{m2}\;\;\cdots \;\;d_{mm} \end{array}\right]$$

(13) $$c_{i,j}=\left[\begin{array}{l} c_{11}\;\;\;\;c_{12}\;\;\;\;\cdots \;\;\;\;c_{1m}\\ c_{21}\;\;\;\;c_{22}\;\;\;\;\cdots \;\;\;\;c_{2m}\\ \;\;\vdots \;\;\;\;\;\;\;\vdots \;\;\;\;\ddots \;\;\;\;\;\;\;\;\vdots \\ c_{m1}\;\;\;\;c_{m2}\;\;\cdots \;\;c_{mm} \end{array}\right]$$

Assuming that the total material–handling cost between each work unit is $F_{1}$, the material–handling cost function of each work unit in the workshop can be obtained according to the material flow and distance:

(14) $$F_{1}=\sum_{i=1}^{m}\sum_{j=1}^{m}c_{i,j}f_{i,j}d_{i,j}$$

The correlation factor proposed by Lee is used to calculate the non–logistic relationship, and the objective function to maximize the non–logistic relationship is constructed [31]. $k_{i,j}$ represents the association factor between units of work i and j. $T_{i,j}$ represents the level of strength of the non–logistic relationship between units of work i and j. The meanings and values of $k_{i,j}$ and $T_{i,j}$ are shown in Table 2 and Table 3. The sum of non–logistic relations can be calculated as follows:

(15) $$F_{2}=\sum_{i=1}^{m}\sum_{j=1}^{m}T_{i,j}k_{i,j}$$

In order to facilitate the solution, the two objective functions are converted into one objective function by increasing the weights $\omega_{1}$ and $\omega_{2}$, as follows:

(16) $$\min F=\omega_{1}\sum_{i=1}^{m}\sum_{j=1}^{m}c_{i,j}f_{i,j}d_{i,j}-\omega_{2}\sum_{i=1}^{m}\sum_{j=1}^{m}T_{i,j}k_{i,j}$$

(17) $${F_{1}}^{`}=\frac{\sum_{i=1}^{m}\sum_{j=1}^{m}c_{i,j}f_{i,j}d_{i,j}}{\sum_{i=1}^{m}\sum_{j=1}^{m}c_{i,j}f_{i,j}d_{\max}}$$

(18) $${F_{2}}^{`}=\frac{\sum_{i=1}^{m}\sum_{j=1}^{m}T_{i,j}k_{i,j}}{\sum_{i=1}^{m}\sum_{j=1}^{m}T_{i,j}}$$

Material–handling costs are just as important as non–logistics relationships, so $\omega_{1}$ and $\omega_{2}$ are each, ultimately, valued at 1. Thus, the final function is updated as follows:

(19) $$\min F={F_{1}}^{`}-{F_{2}}^{`}$$

When using the method of constructing a mathematical model to solve the production workshop problem, it is necessary that we constrain the parameters according to the specifications and dimensions of the designed workshop, so as to obtain a practical layout scheme. For example, it should be ensured that there are no overlapping areas between work units and that work units do not exceed the scope covered by the workshop. First, when considering the mathematical model of the workshop layout optimization problem, it should be ensured that the layout of each work unit does not overlap, and the constraint expression is as follows:

(20) $$\left|x_{i}-x_{j}\right|\geq \frac{L_{i}+L_{j}}{2}+\Delta x_{ij}$$

(21) $$\left|y_{i}-y_{j}\right|\geq \frac{W_{i}+W_{j}}{2}+\Delta y_{ij}$$

**Figure 2:**
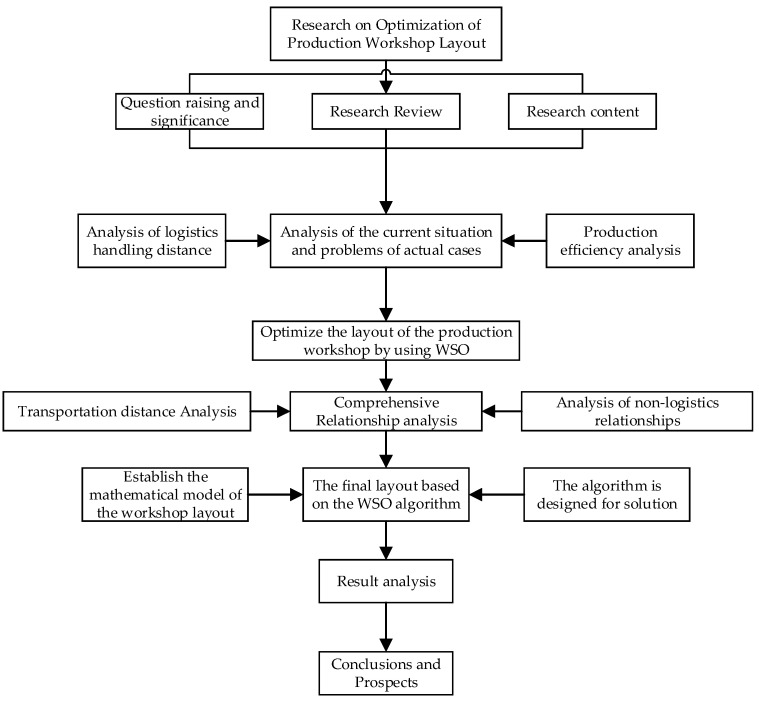
Flowchart based on the proposed method.

All work units should be located on the production floor and subject to certain boundary restrictions. The equation is as follows:

(22) $$\left|x_{i}-x_{j}\right|+\frac{L_{i}+L_{j}}{2}\leq L$$

(23) $$\left|y_{i}-y_{j}\right|+\frac{W_{i}+W_{j}}{2}\leq W$$

where L and W represent the length and width of the workshop, respectively. $L_{i}$ and $W_{i}$ represent the length and width of the work unit i. $L_{j}$ and $W_{j}$ represent the length and width of the work unit j. $\Delta x_{ij}$ and $\Delta y_{ij}$ represent the horizontal and vertical distances between work units i and j, respectively. The flowchart based on the proposed method is shown in Figure 2.

## 4. Experimental Results and Discussion

### 4.1. Case Study

**Figure 3:**
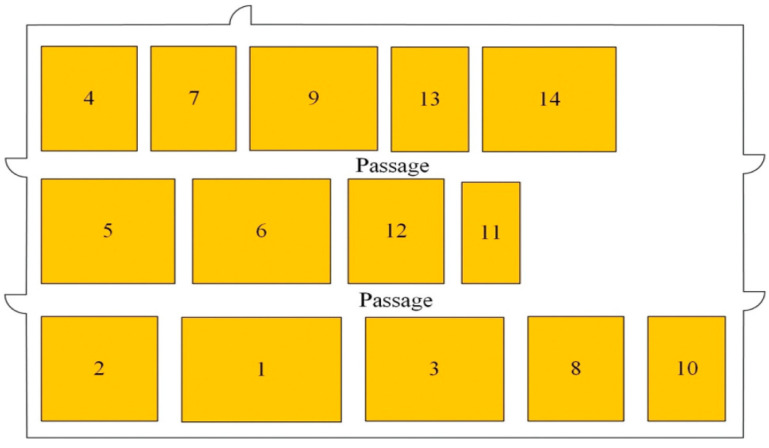
Original layout of the workshop.

All algorithms in this experiment were programmed in MATLAB R2021b, and numerical experiments were carried out on an AMD Ryzen 7 6800H processor with 32 GB of memory. In this chapter, the case in article [32] is used to solve the problem with the White Shark algorithm, and the experimental results are compared and analyzed. In this case, the workshop area is about 3750 square meters, and the workshop length is about 75 m and the width is about 50 m. The workshop is divided into 14 work units, which are as follows: the raw material area, parts area, cutting area, welding area, numerical control area, machining area, bending area, heat treatment area, polishing area, painting area, waste area, semi–finished product area, assembly area, and finished product area. Each work unit in the workshop is numbered, its size table is shown in Table 4, and the original layout of the work unit in the workshop is shown in Figure 3. There are two passageways 6 m wide in the workshop, with a minimum spacing of 3 m between each work unit and a distance of 1 m between the work unit and the edge of the workshop. The values of material flow and non–logistic relations in the workshop are shown in Table 5 and Table 6. The mathematical model aims to minimize material–handling costs and maximize non–logistical relationships, with the objective function shown in Equation (19).

### 4.2. Analysis and Comparison

**Figure 4:**
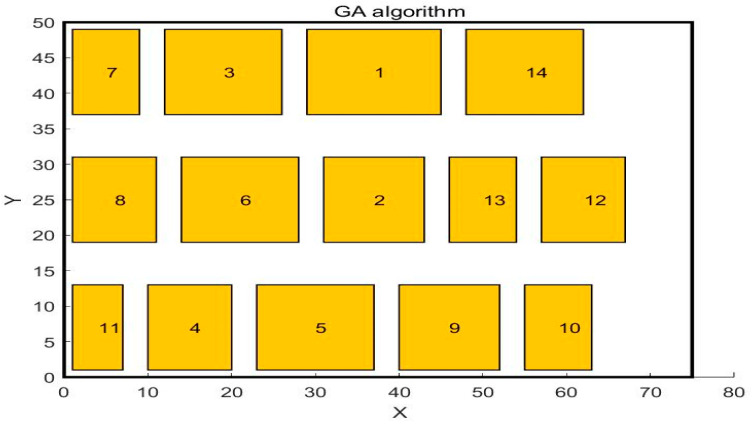
Layout diagram after GA algorithm optimization.

**Figure 5:**
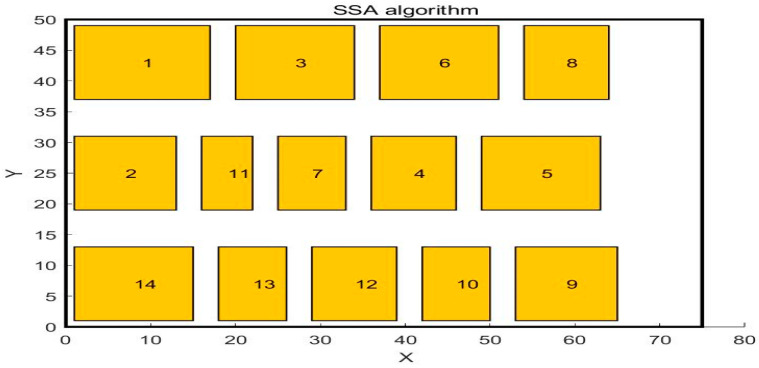
Layout diagram after SSA algorithm optimization.

**Figure 6:**
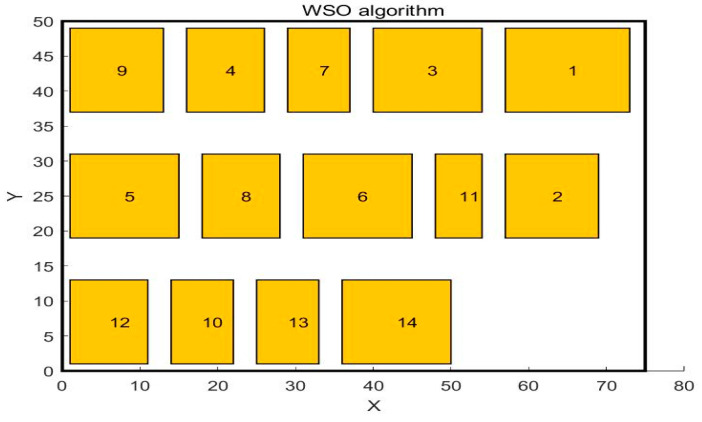
Layout diagram after WSO algorithm optimization.

In the paper [32], the author adopted the SSA algorithm to solve the problem and compared it with the GA algorithm. The workshop layout diagram obtained by the GA algorithm is shown in Figure 4, and the workshop layout diagram obtained by the SSA algorithm is shown in Figure 5. This paper uses WSO to solve this problem. The central point coordinates of the work units obtained by WSO to solve this problem are shown in Table 7, and the distribution of each work unit in the workshop is shown in Figure 6. The scheme obtained by the WSO algorithm is compared with the original workshop layout scheme, the GA algorithm, and the SSA algorithm.

**Figure 7:**
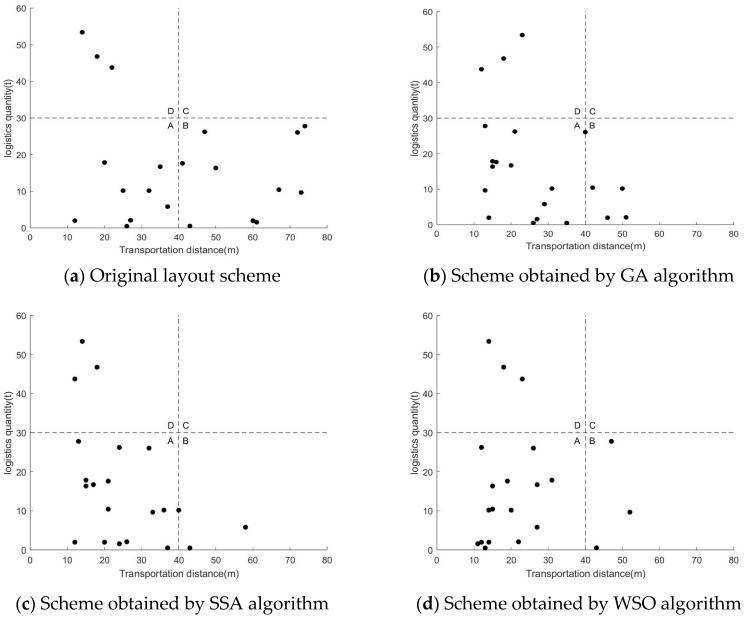
Logistics quantity–transportation distance.

First of all, the logistics transportation distance between each work unit in the workshop is compared. The comparison of transport distances between work units is shown in Table 8, where the pair of work units represents the logistics transport between two work units. Table 8 shows that the total processing distance of the original workshop layout scheme, the GA and SSA algorithms, and the solutions obtained by the WSO algorithm are 856 m, 557 m, 531 m, and 475 m, respectively. The total transportation distance of the WSO algorithm is the shortest, which is 381 m shorter than the original workshop layout scheme, 82 m shorter than the GA algorithm, and 56 m shorter than the SSA algorithm. In order to intuitively show the logistics relationship between each work unit, this paper draws an F–D diagram, as shown in Figure 7. The F–D chart is divided into four areas, with the vertical axis representing the volume of goods and the horizontal axis representing the distance transported. The points in Zone A represent a small flow rate and a short transportation distance, which is very beneficial to the production of the entire workshop. The point in Zone B represents a long transportation distance and a small flow rate, which is unfavorable to the production of the workshop. Zone C represents a long transportation distance and a large flow of goods, which is very unfavorable to production. Zone D represents a short transportation distance and a large volume of goods, which is a more reasonable situation. The layout of the workshop should reduce the number of working units in Zone B and Zone C, which is beneficial to the production development of the workshop. It can be seen from Figure 7 that the WSO and SSA algorithms obtained three pairs of working units in B and C, while the GA algorithm obtained five pairs of working units in B and C, and the original layout obtained ten pairs of working units in B and C. This shows that the workshop layout obtained by the WSO algorithm is reasonable.

**Figure 8:**
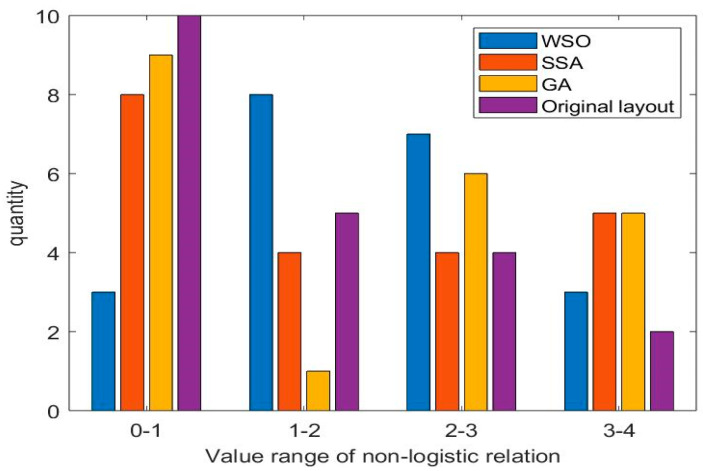
Graph of non–logical relationship values.

Table 9 shows the non–logistic relationship between the work unit pairs obtained by different algorithms. The larger the value of the unit pairs, the better the non–logical relationship between the two work units. The non–logistic relationship values obtained by the original layout, the GA algorithm, the SSA algorithm and the WSO algorithm are 30.6, 37.6, 38.2, and 38.2, respectively. Among the four layout schemes, the WSO algorithm and the SSA algorithm obtained the largest non–logistics relationship value. It is 24.84% higher than the original workshop layout scheme and 1.60% higher than the scheme obtained by the GA algorithm. This verifies the rationality of the layout scheme obtained by the WSO algorithm. A bar chart is drawn to more intuitively show the non–logistic relationships between work units, as shown in Figure 8. The horizontal coordinate represents the value range of the non–logistic relationship, and the vertical coordinate represents the number of non–logistic relationship values within the specified interval. As can be seen from Figure 8, the non–logistical relationships range between 0 and 1, and the number of solutions obtained by WSO is the smallest within this range. In the 1 to 2 and 2 to 3 ranges, WSO received the largest number of proposals. On the whole, the non–logistic relationship between the pairs of work units in the layout scheme obtained by the WSO solution is strong.

### 4.3. Case Development

**Figure 9:**
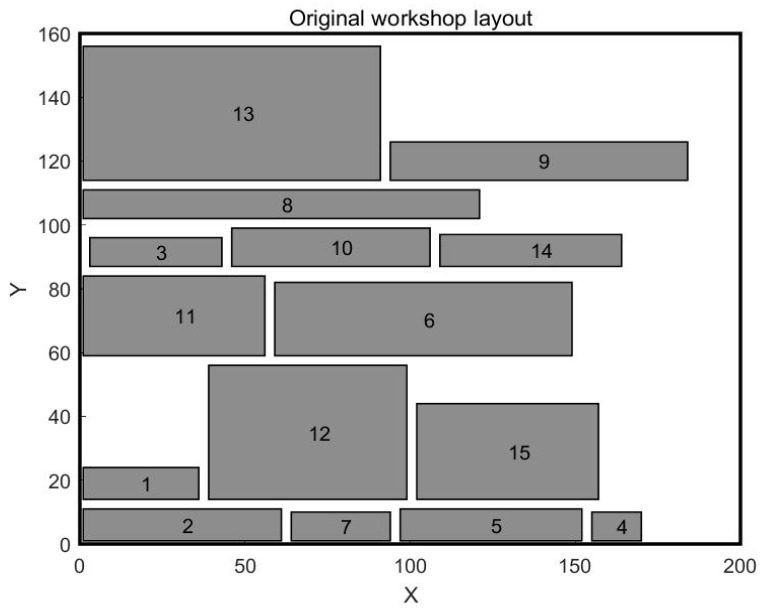
Original workshop layout for Case 2.

**Figure 10:**
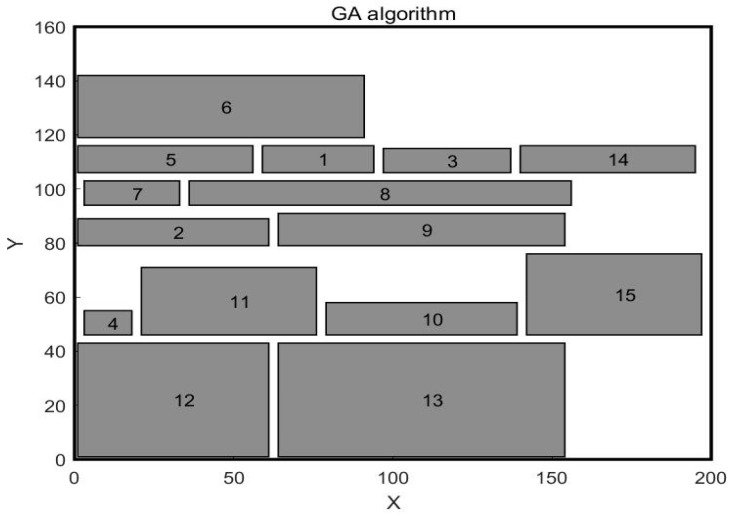
Workshop layout after GA optimization.

**Figure 11:**
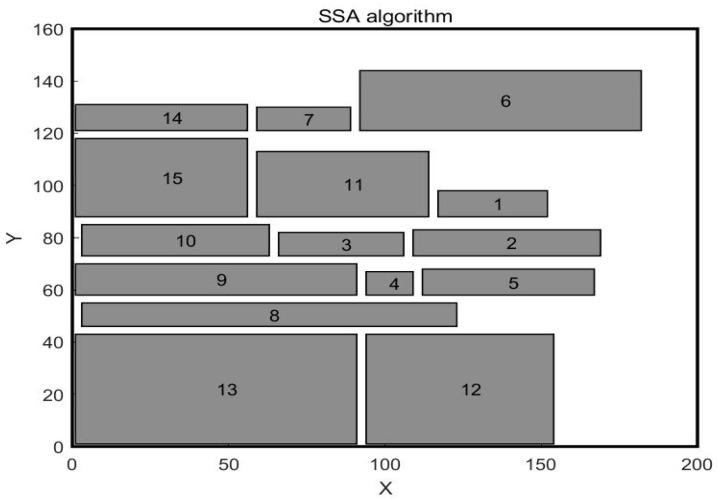
Workshop layout after SSA optimization.

In the previous case, the units of work were equal in width and less different in length. In order to further verify the effectiveness of the White Shark algorithm, the second case is selected for study, which is selected from the article [32]. In this case, the workshop is about 200 m long and 160 m wide, and the workshop is divided into 15 work units. The area differences between work units are significant, with the length and width parameters of each work unit shown in Table 10. The original workshop layout is illustrated in Figure 9, while the layouts obtained by the Genetic Algorithm and the Sparrow Algorithm are presented in Figure 10 and Figure 11, respectively. The mathematical model is still aimed at minimizing material–handling costs and maximizing non–logistical relationships. The values of material flow and non–logistic relations in the workshop are shown in Table 11 and Table 12.

**Figure 12:**
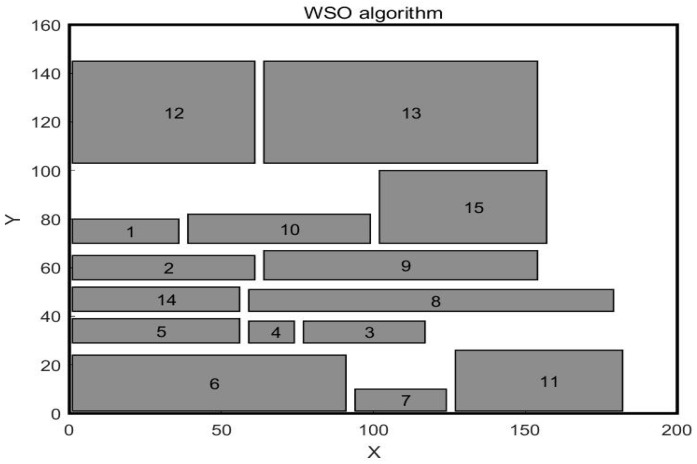
Workshop layout after WSO optimization.

The WSO algorithm is used to optimize the workshop layout in Case 2. The resulting layout scheme is shown in Figure 12. The comparative analysis results of the optimized logistics transportation distance and non–logistics relationship are shown in Table 13 and Table 14. As can be seen from Table 13, the total material transport distance of the original layout scheme of Case 2, the scheme obtained by the GA algorithm, the solution obtained by the SSA algorithm, and the solution obtained by the WSO algorithm is 1610 m, 1099.5 m, 837.5 m, and 815.5 m, respectively. Among all the results, the total material transport distance obtained by the WSO algorithm is still the best. It is 805 m shorter than the original workshop layout scheme, 284 m shorter than the solution obtained by the GA algorithm, and 22 m shorter than the solution obtained by the SSA algorithm. In Case 2, the total scores of non–logical relations of the original layout scheme, the scheme obtained by the GA algorithm, the scheme obtained by the SSA algorithm, and the scheme obtained by the WSO algorithm are 30.6, 34.2, 35.8, and 36, respectively. Among the original layout scheme and the optimized layout scheme obtained by the three algorithms, the scheme obtained by the WSO algorithm has the highest total non–logical relation score. It is 17.6% higher than the original workshop layout plan, 5.3% higher than the solution obtained by the GA algorithm, and 0.6% higher than the solution obtained by the SSA algorithm. Through the optimization research on the workshop layout of Case 2, the WSO algorithm performs best in both the transportation distance of logistics and the non–logistics relationship between work units, further verifying the superiority and effectiveness of the WSO algorithm.

The Spearman correlation coefficient is a non–parametric statistic used to measure the strength of the monotonic relationship between two variables. This paper selects the values between the logistics transportation distance and the non–logistics relationship under 10 different layout situations, and uses the Spearman correlation coefficient to evaluate the relationship between the logistics transportation distance and the non–logistics relationship. The calculation results are shown in Table 15. Through calculation, the Spearman correlation coefficient between the two groups of data is −0.9424. This value is close to −1, indicating that there is a strong negative correlation between the two groups of data. That is, with the increase in the transportation distance value, the value of the non–logistics relationship decreases significantly. Specifically, the extreme value of the transportation distance corresponds to the lowest value of the non–logistics relationship, while the smaller value of the transportation distance corresponds to the higher value of the non–logistics relationship, indicating a significant monotonically decreasing trend between the transportation distance and the non–logistics relationship.

## 5. Conclusions

This paper investigates workshop layout optimization using the White Shark Optimizer (WSO) algorithm. The objective functions for the workshop layout problem are defined as minimizing logistics transportation costs and maximizing non–logistics relationships. The WSO algorithm is employed to solve the mathematical model, resulting in an optimized workshop layout plan. The layout outcomes obtained by the WSO algorithm are compared with the original layout and those derived from other algorithms. The analysis demonstrates the effectiveness of the WSO algorithm. In Case 1, the transportation distance of the layout scheme obtained by the WSO algorithm was reduced by 381 m, 82 m, and 56 m, respectively, compared with the original layout, the Genetic Algorithm, and Sparrow Algorithm, and the non–logical relationship was increased by 24.84% and 1.6%, respectively. In Case 2, the transportation distance of the layout scheme obtained by the WSO algorithm was reduced by 805 m, 284 m, and 22 m, respectively, compared with the original layout, the Genetic Algorithm, and the Sparrow Algorithm, and the non–logical relationship was increased by 17.6%, 5.3%, and 0.6%, respectively. This indicates that the workshop layout scheme obtained by the WSO algorithm can effectively improve the production efficiency of enterprises and has certain scientific theoretical significance.

This study has limitations, which need to be addressed in subsequent studies. Firstly, in terms of mathematical modeling, although the two core elements of the workshop logistics relationship and non–logistics relationship were comprehensively considered, development indicators such as environmental benefits were not fully taken into account. With the transformation and development of the manufacturing industry, it is suggested that subsequent studies consider integrating environmental variables such as energy consumption, waste treatment, and carbon emission intensity into mathematical models. Secondly, in the future, hybrid solution strategies such as deep learning can be combined to improve meta–heuristic algorithms.

## Figures and Tables

**Table 1:** Comparison of various methods.

Reference	Factors Considered	Results Obtained	Positive Sides	Limitations	Scope for Further Study
Fahad et al. [19]	Environmental and economic goals	Improve transportation efficiency and reduce lighting energy consumption	Achieve a win–win situation for the economy and environmental protection	The actual scenarios such as equipment failure and workers’ efficiency were not taken into consideration	Establish a cost–benefit model to provide enterprises with a basis for economic decision–making.
Bhuvanesh et al. [20]	Transportation distance and waiting time	Shorten the production cycle and reduce the transportation distance	Simplify the framework and optimize the production process	Lack of economic analysis and reliance on experts to allocate weights	Introduce multi–method comparison
E–Baz. [21]	Production volume, transportation route, and equipment spacing	The algorithm performs stably and effectively reduces the handling cost	Algorithm innovation enhances search capabilities	Large–scale problems take a longer time	Introduce multi–objective optimization and construct a comprehensive optimization model
Su Sd et al. [22]	Take the circulation intensity and adjacency intensity of the cabin as the core objectives	Significantly improve circulation efficiency and functional adjacency	Introduce the SLP method for solving factory layout into ship design	Practical constraints such as weight distribution and equipment installation were not taken into account	Apply the method to complex ship types such as industrial ships to verify their universality
Nguyen et al. [23]	Actual constraint conditions, and multi–objective optimization	Verify through cases that the algorithm performs well in terms of cost and other aspects	It balanced multiple conflicting targets and provided a reference plan for the construction layout	The complex factors of noise attenuation were not considered	Explore the optimization ability of the algorithm in the dynamic construction environment
Gonzalez–Cruz et al. [24]	Consider the main attributes and the relationships among various elements	The entropy algorithm performs better in aspects such as reducing transportation distances	Introduce the concept of entropy into the optimization of facility layout	This algorithm has not been extended to the actual scenarios in two–dimensional or three–dimensional space at present	Test the universality of the algorithm in complex industrial scenarios
Garcia–Hernandez et al. [25]	With the material flow cost as the core	The algorithm performs well in small– and medium–sized instances	It has the potential for expansion in multi–objective optimization and dynamic scenarios	The performance of the algorithm depends on the adjustment of empirical parameters	Explore the variants of the algorithm to optimize the convergence speed and global search ability
Korde et al. [26]	Limitations of physical space and equipment size	Reduce the distance of logistics transportation and lower costs	The validity of the case verification method can be extended to actual scenarios	Ignore the randomness of actual production	Multi–dimensional indicators are considered in the design to achieve comprehensive optimization

**Table 2:** Correlation factor quantification.

Distance Between Work Units $d_{i,j}$	$k_{i,j}$
$0<d_{i,j}\leq d_{\max}/6$	1
$d_{\max}/6<d_{i,j}\leq d_{\max}/3$	0.8
$d_{\max}/3<d_{i,j}\leq d_{\max}/2$	0.6
$d_{\max}/2<d_{i,j}\leq 2d_{\max}/3$	0.4
$2d_{\max}/3<d_{i,j}\leq 5d_{\max}/6$	0.2
$5d_{\max}/6<d_{i,j}\leq d_{\max}$	0

**Table 3:** Quantified level of non–logistics closeness.

Non–Logistics Level	Expressing Relationship	$T_{i,j}$
A	Extremely desirable	4
E	Very desirable	3
I	Desirable	2
O	Indifferent	1
U	Unimportant	0
X	Undesirable	−1

**Table 4:** Parameters of each unit in the workshop.

No	Work Unit Name	Length (m)	Width (m)
1	Raw material area	16	12
2	Parts area	12	12
3	Cutting area	14	12
4	Welding area	10	12
5	CNC area	14	12
6	Machining area	14	12
7	Bending area	8	12
8	Heat treatment area	10	12
9	Polishing area	12	12
10	Painting area	8	12
11	Waste area	6	12
12	Semi–finished product area	10	12
13	Assembly area	8	12
14	Finished product area	14	12

**Table 5:** Logistics quantity between the two work units.

Unit	1	2	3	4	5	6	7	8	9	10	11	12	13	14
1	0	0	46.77	0	0	0	0	0	0	0	0	0	0	1
2	0	0	0	0	0	0	0	0	0	0	0	0	9.651	2
3	0	0	0	26.049	0	16.689	1.965	0	0	0	2.067	0	0	3
4	0	0	0	0	17.841	0	0	0	10.173	0	0	0	0	4
5	0	0	0	0	0	0	0	0	17.601	0	0.51	10.158	0	5
6	0	0	0	0	0	0	0	16.338	0	0	0.468	0	5.793	6
7	0	0	0	1.965	0	0	0	0	0	0	0	0	0	7
8	0	0	0	0	10.428	5.91	0	0	0	0	0	0	0	8
9	0	0	0	0	0	0	0	0	0	27.774	0	0	0	9
10	0	0	0	0	0	0	0	0	0	0	0	26.223	1.551	10
11	0	0	0	0	0	0	0	0	0	0	0	0	0	11
12	0	0	0	0	0	0	0	0	0	0	0	0	43.746	12
13	0	0	0	0	0	0	0	0	0	0	0	7.365	0	13
14	0	0	0	0	0	0	0	0	0	0	0	0	0	14

**Table 6:** Value of non–logistic relationship between the two work units.

Unit	1	2	3	4	5	6	7	8	9	10	11	12	13	14
1	0	0	4	0	0	0	0	0	0	0	0	0	0	0
2	0	0	0	0	0	0	0	0	0	0	0	0	2	0
3	0	0	0	3	0	3	2	0	0	0	1	0	0	0
4	0	0	0	0	2	0	0	0	1	0	0	0	0	0
5	0	0	0	0	0	0	0	0	2	0	1	1	0	0
6	0	0	0	0	0	0	0	4	0	0	1	0	1	0
7	0	0	0	1	0	0	0	0	0	0	0	0	0	0
8	0	0	0	0	1	4	0	0	0	0	0	0	0	0
9	0	0	0	0	0	0	0	0	0	2	0	0	0	0
10	0	0	0	0	0	0	0	0	0	0	0	2	1	0
11	0	0	0	0	0	0	0	0	0	0	0	0	0	0
12	0	0	0	0	0	0	0	0	0	0	0	0	3	0
13	0	0	0	0	0	0	0	0	0	0	0	3	0	4
14	0	0	0	0	0	0	0	0	0	0	0	0	0	0

**Table 7:** WSO algorithm scheme of work unit center coordinate values.

No	Work Unit Name	X	Y
1	Raw material area	65	43
2	Parts area	63	25
3	Cutting area	47	43
4	Welding area	21	43
5	CNC area	8	25
6	Machining area	38	25
7	Bending area	33	43
8	Heat treatment area	23	25
9	Polishing area	7	43
10	Painting area	18	7
11	Waste area	51	25
12	Semi–finished product area	6	7
13	Assembly area	29	7
14	Finished product area	43	7

**Table 8:** The transport distance between working units obtained by different algorithms (m).

Work Unit Pairs	Original Layout Scheme	Scheme Obtained by GA Algorithm	Scheme Obtained by SSA Algorithm	Scheme Obtained by WSO Algorithm
1–3	18	18	18	18
213	73	13	33	52
3–4	72	40	32	26
3–6	35	20	17	27
3–7	60	14	20	14
3–11	27	51	26	22
4–5	20	15	15	31
4–7	12	46	12	12
4–9	25	31	36	14
5–8	67	42	21	15
5–9	41	16	21	19
5–11	43	26	37	43
5–12	32	50	40	20
6–8	50	15	15	15
6–11	26	35	43	13
6–13	37	29	58	27
9–10	74	13	13	47
10–12	47	21	24	12
10–13	61	27	24	11
12–13	22	12	12	23
13–14	14	23	14	14
Total	856	557	531	475

**Table 9:** Comparison table of non–logistic relations between work units.

Work Unit Pairs	Original Layout Scheme	Scheme Obtained by GA Algorithm	Scheme Obtained by SSA Algorithm	Scheme Obtained by WSO Algorithm
1–3	4	4	4	4
2–13	0.8	2	1.6	1.2
3–4	1.2	2.4	2.4	2.4
3–6	2.4	3	3	2.4
3–7	1.2	2	2	2
3–11	0.8	0.6	0.8	0.8
4–5	2	2	2	1.6
4–7	1	0.6	1	1
4–9	0.8	0.8	0.8	1
5–8	0.4	0.6	0.8	1
5–9	1.6	2	1.6	2
5–11	0.6	0.8	0.8	0.6
5–12	0.8	0.6	0.8	1
6–8	2.4	4	4	4
6–11	0.8	0.8	0.6	1
6–13	0.8	0.8	0.6	0.8
9–10	0.8	2	2	2
10–12	1.2	1.6	1.6	2
10–13	0.6	0.8	0.8	1
12–13	2.4	3	3	2.4
13–14	4	3.2	4	4
Total	30.6	37.6	38.2	38.2

**Table 10:** Unit of work parameters for Case 2.

No	Length (m)	Width (m)
1	35	10
2	60	10
3	40	9
4	15	9
5	55	10
6	90	23
7	30	9
8	120	9
9	90	12
10	60	12
11	55	25
12	60	42
13	90	42
14	55	10
15	55	30

**Table 11:** Logistics quantity between the two work units in Case 2.

Work	1	2	3	4	5	6	7	8	9	10	11	12	13	14	15
1	0	31.58	0	0	0	0	0	0	0	0	0	0	0	0	0
2	0	0	0	0	28.21	0	0	0	0	0	0	0	0	0	0
3	0	0	0	11.87	0	0	0	0	0	0	0	0	0	0	0
4	0	0	0	0	10.53	0	0	0	0	0	0	0	0	0	0
5	0	0	0	0	0	38.41	0	0	0	0	0	0	0	0	0
6	0	0	0	0	0	0	38.4	0	0	0	0	0	0	0	0
7	0	0	0	0	0	0	0	6.3	8.06	0	24.05	0	0	0	0
8	0	0	0	0	0	0	0	0	35.39	0	0	0	0	0	0
9	0	0	0	0	0	0	0	0	0	43.45	0	0	0	0	54.8
10	0	0	0	0	0	0	0	0	0	0	0	0	43.3	0	0
11	0	0	0	0	0	0	0	46.2	0	0	0	0	0	0	0
12	0	0	0	0	0	0	0	0	0	0	0	0	11.5	0	0
13	0	0	0	0	0	0	0	0	54.8	0	0	0	0	0	0
14	0	0	0	0	0	0	0	0	0	0	0	0	0	0	0
15	0	0	0	0	0	0	0	0	0	0	0	0	0	0	0

**Table 12:** Value of non–logistic relationship between the two work units in Case 2.

Work	1	2	3	4	5	6	7	8	9	10	11	12	13	14	15
1	0	2	0	0	0	0	0	0	0	0	0	0	0	0	0
2	0	0	0	0	2	0	0	0	0	0	0	0	0	0	0
3	0	0	0	2	0	0	0	0	0	0	0	0	0	0	0
4	0	0	0	0	1	0	0	0	0	0	0	0	0	0	0
5	0	0	0	0	0	3	0	0	0	0	0	0	0	0	0
6	0	0	0	0	0	0	3	0	0	0	0	0	0	0	0
7	0	0	0	0	0	0	0	1	1	0	2	0	0	0	0
8	0	0	0	0	0	0	0	0	3	0	4	0	0	0	0
9	0	0	0	0	0	0	0	0	0	3	0	0	4	0	4
10	0	0	0	0	0	0	0	0	0	0	0	0	3	0	0
11	0	0	0	0	0	0	0	0	0	0	0	0	0	0	0
12	0	0	0	0	0	0	0	0	0	0	0	0	1	0	0
13	0	0	0	0	0	0	0	0	0	0	0	0	0	0	0
14	0	0	0	0	0	0	0	0	0	0	0	0	0	0	0
15	0	0	0	0	0	0	0	0	0	0	0	0	0	0	0

**Table 13:** The transport distance between working units obtained by different algorithms in Case 2 (m).

Work Unit Pairs	Original Layout Scheme	Scheme Obtained by GA Algorithm	Scheme Obtained by SSA Algorithm	Scheme Obtained by WSO Algorithm
1–2	25.5	72.5	19.5	27.5
2–5	93.5	29.5	15.5	28.5
3–4	225.5	166.5	30.5	30.5
4–5	38.5	78.5	38.5	38.5
5–6	85	37	72	39
6–7	90	60	70	70
7–8	119	78	86	51
7–9	174.5	104.5	89.5	55.5
7–11	116.5	70.5	37.5	53.5
8–9	91.5	26.5	30.5	24.5
8–11	67.5	87.5	73.5	68.5
9–10	90	33	28	55
9–13	108	63	42	63
9–15	100.5	84.5	56.5	44.5
10–13	72	30	70	88
12–13	123	78	78	78
Total	1610	1099.5	837.5	815.5

**Table 14:** Comparison table of non–logistic relations between work units in Case 2.

Work Unit Pairs	Original Layout Scheme	Scheme Obtained by GA Algorithm	Scheme Obtained by SSA Algorithm	Scheme Obtained by WSO Algorithm
1–2	2	1.6	2	2
2–5	1.6	2	2	2
3–4	0.8	1.2	2	2
4–5	1	0.8	1	1
5–6	2.4	3	2.4	3
6–7	2.4	3	2.4	2.4
7–8	0.8	0.8	0.8	1
7–9	0.6	0.8	0.8	1
7–11	1.6	1.6	2	2
8–9	2.4	3	3	3
8–11	3.2	3.2	3.2	3.2
9–10	2.4	3	3	3
9–13	3.2	3.2	4	3.2
9–15	3.2	3.2	4	4
10–13	2.4	3	2.4	2.4
12–13	0.6	0.8	0.8	0.8
Total	30.6	34.2	35.8	36

**Table 15:** The rank table between logistics transportation distance and non–logistics relationship.

Layout	Transportation Distance	Non–Logistics Relationship	Rank_Transportation Distance	Rank_Non–Logistics Relationship	$d_{i}$	$d_{i}^{2}$
1	815.5	36	1	8.5	−7.5	56.25
2	836.5	36.6	2	10	−8	64
3	837.5	35.8	3	7	−4	16
4	893.5	36	4	8.5	−4.5	20.25
5	967.5	35.6	5	6	−1	1
6	989.5	34.8	6	4	2	4
7	1002.5	35	7	5	2	4
8	1034.5	34.6	8	3	5	25
9	1099.5	34.2	9	2	7	49
10	1610	30.6	10	1	9	81

## Data Availability

The data presented in this study are available upon request from the corresponding author.
